# Plectin Deficiency Leads to Both Muscular Dystrophy and Pyloric Atresia in Epidermolysis Bullosa Simplex

**DOI:** 10.1002/humu.21330

**Published:** 2010-10

**Authors:** Ken Natsuga, Wataru Nishie, Satoru Shinkuma, Ken Arita, Hideki Nakamura, Makiko Ohyama, Hitoshi Osaka, Takeshi Kambara, Yoshiaki Hirako, Hiroshi Shimizu

**Affiliations:** 1 Department of Dermatology, Hokkaido University Graduate School of MedicineSapporo, Japan; 2 Department of Pediatrics, Kanagawa Children's Medical CenterYokohama, Japan; 3 Department of Neurology, Kanagawa Children's Medical CenterYokohama, Japan; 4 Department of Dermatology, Yokohama City University School of MedicineYokohama, Japan; 5 Division of Biological Science, Graduate School of Science, Nagoya UniversityNagoya, Japan

**Keywords:** basement membrane zone, skeletal muscle, mRNA decay, truncation

## Abstract

Plectin is a cytoskeletal linker protein which has a long central rod and N- and C-terminal globular domains. Mutations in the gene encoding plectin (*PLEC*) cause two distinct autosomal recessive subtypes of epidermolysis bullosa: EB simplex (EBS) with muscular dystrophy (EBS-MD), and EBS with pyloric atresia (EBS-PA). Previous studies have demonstrated that loss of full-length plectin with residual expression of the rodless isoform leads to EBS-MD, whereas complete loss or marked attenuation of expression of full-length and rodless plectin underlies the more severe EBS-PA phenotype. However, muscular dystrophy has never been identified in EBS-PA, not even in the severe form of the disease. Here, we report the first case of EBS associated with both pyloric atresia and muscular dystrophy. Both of the premature termination codon-causing mutations of the proband are located within exon 32, the last exon of *PLEC*. Immunofluorescence and immunoblot analysis of skin samples and cultured fibroblasts from the proband revealed truncated plectin protein expression in low amounts. This study demonstrates that plectin deficiency can indeed lead to both muscular dystrophy and pyloric atresia in an individual EBS patient. © 2010 Wiley-Liss, Inc.

## INTRODUCTION

Plectin is a 500-kDa protein of the plakin family, which interlinks different element of the cytoskeleton (Rezniczek, et al., 2010). Plectin is prominently expressed in muscle and in stratified and simple epithelia, including in the skin and gastrointestinal tract (Rezniczek, et al., 2010). In skin, plectin localizes to the inner plaque of the hemidesmosomes, at the site of interaction with intermediate filaments (Smith, et al., 1996). Plectin has a unique dumbbell-like structure with a central rod domain and N- and C-terminal globular domains (Wiche, et al., 1991). Various types of plectin transcripts, including rodless ones, that do not encode for a central rod domain have been reported (Elliott, et al., 1997).

Epidermolysis bullosa (EB) comprises a group of heterogeneous congenital disorders characterized by dermal- epidermal junction separation. EB is subdivided into the three major groups of EB simplex (EBS), junctional EB and dystrophic EB, and the one minor group of Kindler syndrome, based on the level of blister formation (Fine, et al., 2008). So far, mutations in 14 different genes have been identified as underlying the EB subtypes (Fine, et al., 2008; Groves, et al., 2010). Among them, mutations in the gene encoding plectin, *PLEC* (MIM# 601282), have been known to be causal for two subtypes of autosomal recessive EBS (EBS with muscular dystrophy (EBS-MD) and EBS with pyloric atresia (EBS-PA)) and for one subtype of autosomal dominant EBS (EBS-Ogna) (Fine, et al., 2008).

Characteristic manifestations of EBS-MD are generalized skin blistering and late onset muscle weakness. Previous studies revealed defective expression of plectin in EBS-MD skin samples (Gache, et al., 1996; Shimizu, et al., 1999a; Shimizu, et al., 1999b) and mutations in *PLEC* in EBS-MD patients (McLean, et al., 1996; Pulkkinen, et al., 1996; Smith, et al., 1996; Takizawa, et al., 1999). The *PLEC* mutations detected in EBS-MD patients are mainly within exon 31, which encodes the large-rod domain of plectin (Natsuga, et al., 2010; Pfendner, et al., 2005; Sawamura, et al., 2007).

In contrast to patients with EBS-MD, those with EBS-PA typically develop a more severe phenotype that includes more generalized blistering and pyloric atresia (PA) (Nakamura, et al., 2005). The prognosis of EBS-PA is very poor, and affected patients usually die within months after birth (Nakamura, et al., 2005; Pfendner, et al., 2005; Pfendner and Uitto, 2005). *PLEC* mutations of EBS-PA were mostly located outside of exon 31 (Natsuga, et al., 2010).

Although both EBS-MD and EBS-PA are autosomal recessive EBS caused by *PLEC* mutations, the pathomechanisms distinguishing two subtypes were unclear. Recently, our group and others demonstrated that EBS-MD patients typically express a rodless plectin isoform, although the full-length plectin is absent (Koster, et al., 2004; Natsuga, et al., 2010). In contrast, both full-length and rodless plectin isoforms are deficient in EBS-PA patients, leading to the more severe disease phenotype (Natsuga, et al., 2010). In light of these findings, it has been postulated that EBS-PA patients could develop muscular dystrophy (MD) if they survived longer (Natsuga, et al., 2010). However, to our knowledge, there have been no EBS patients who suffered from both MD and PA.

Here, we report the first patient with EBS who developed both PA and MD. Both of the mutations identified in the patient were within the last exon (exon 32) of *PLEC*. Immunofluorescence and immunoblot analysis confirmed diminished and truncated plectin expression, using several antibodies against different domains of plectin. This study gives further insight toward improving our understanding of the genotype-phenotype correlation in EBS patients with *PLEC* mutations.

## MATERIALS AND METHODS

### Electron Microscopy

Skin biopsy samples were fixed in 2% glutaraldehyde solution, post-fixed in 1% OsO_4_, dehydrated, and embedded in Epon 812. The samples were sectioned at 1 um thickness for light microscopy and thin-sectioned for electron microscopy (70 nm thick). The thin sections were stained with uranyl acetate and lead citrate, and examined by transmission electron microscopy.

### Mutation Detection

Genomic DNA (gDNA) was isolated from peripheral blood leukocytes of the proband and her parents. The mutation detection was performed after polymerase chain reaction (PCR) amplification of all *PLEC* exons and intron-exon borders, followed by direct automated sequencing using an ABI PRISM 3100 genetic analyzer (Applied Biosystems, Foster City, CA). The oligonucleotide primers and PCR conditions used in this study were derived from a previous report (Nakamura, et al., 2005). The gDNA nucleotides, the complementary DNA (cDNA) nucleotides and the amino acids of the protein, were numbered based on the GenBank sequence information (accession no. NM_000445.3 ). PCR amplification of two parts of exon 32 was performed using the following primers. Primers 5′-GTGGAGACCACGCAGGTGTAC-3′ and 5′-GGAGCCCGTGCCATAGAGG-3′ for a single part of exon 32 synthesized a 420-bp fragment including c.10735 to c.11154. Primers 5′- AGCGGCTGACTGTGGATGAGG-3′ and 5′-TGCGTGTCCTTGTTGAGGT-3′ for another single part of exon 32 synthesized a 283-bp fragment including c. 11230 to c. 11512. Both of the mutations in the proband were confirmed by restriction digestion of PCR products. c.10984C>T and c.11453_11462del caused the generation of new restriction enzyme sites for *BsrI* and *BbvCI,* respectively.

The mutation nomenclature follows the journal's guidelines (http://www.hgvs.org/mutnomen) according to the reference sequence NM_000445.3, with +1 as the A of the ATG initiation codon.

### Haplotype analysis

Genotype analysis of this family to establish the *de novo* nature of c.11453_11463del in the proband was performed using three chromosome 8 markers (D8S272, D8S264, D8S270) and six non-chromosome 8 markers (D1S468, D1S252, D1S2842, D3S1297, D3S1566 and D3S1311). All microsatellite markers (ABI Prism Linkage Mapping Set Version 2.5; Applied Biosystems, Warrington, UK) were amplified with fluorescently labeled oligonucleotides and used under conditions recommended by the manufacturer. Electrophoretic analysis was performed on an ABI Prism 310 Genetic Analyzer with Performance Optimized Polymer 4 (POP4) using GeneScan software (Applied Biosystems). The allele sizes were analyzed using Genotyper software (Applied Biosystems).

### Immunofluorescence Studies

Immunofluorescence analysis was performed using skin specimens from the proband as previously described (Natsuga, et al., 2010). Briefly, fresh-frozen skin specimens were embedded in optimal cutting temperature (OCT) compound and quickly frozen in isopentane cooled over liquid nitrogen. 5-μm cryostat sections were incubated with primary antibodies. After washing in phosphate-buffered saline, the sections were incubated with secondary antibodies conjugated with fluorescein-isothiocyanate.

### Antibodies

**Figure 1:**
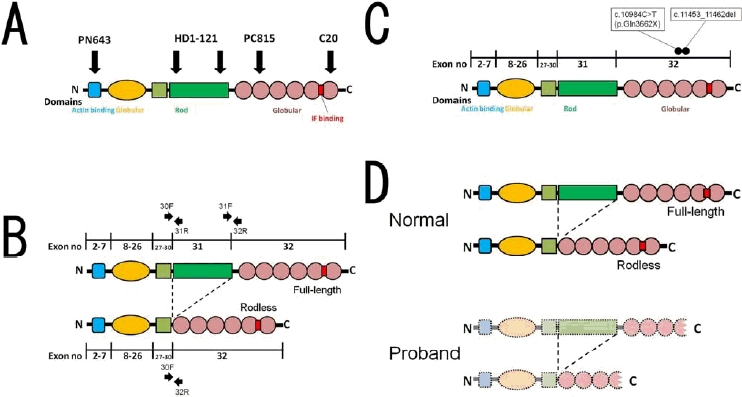
Plectin structure, antibodies against plectin, specific primers to amplify the full-length and the rodless plectin transcripts and *PLEC* mutations of the proband. (A) Plectin protein is composed of an actin-binding domain, N- and C-terminal globular domains, an intermediate filament (IF)-binding domain and a central rod domain. The C-terminal globular domain has 6 plectin repeat domains. The IF-binding domain is located between C-terminal repeats 5 and 6. PN643 is a monoclonal antibody (mAb) against the N-terminal actin-binding domain of plectin. HD1-121 is a mAb against the rod domain of plectin. PC815 is a mAb and C20 is a polyclonal antibody against the C-terminal globular domain of plectin. (B) The specific primers used to detect the presence of transcripts for full-length (30F/31R and 31F/32R) and rodless plectin (30F/32R) on cDNA synthesized from mRNA of normal humans and the proband's fibroblasts. (C) c.10984C>T and c.11453_11462del are located in the *PLEC* encoding C-terminal plectin repeat 4. (D) Normal humans express both full-length and rodless plectin. In our case, the *PLEC* mutations produced diminished and truncated plectin protein without the IF-binding domain.

The following antibodies against basement membrane zone (BMZ) components were used: monoclonal antibody (mAb) PN643 against the N-terminal actin-binding domain of plectin; mAb HD1-121 against the rod domain of plectin; C20 and mAb PC-815 against the C-terminal globular domain of plectin (Fig. 1A); mAbs GoH3 and 3E1 (Chemicon International, CA) against α6 and ß4 integrins, respectively; mAb GB3 (Sera-lab, Cambridge, UK) against laminin 332; mAb LH7.2 (Sigma, St. Louis, MO) against type VII collagen; mAb PHM-12^+^CIV22 against type IV collagen (NeoMarkers, Fremont, CA); and S1 193 and mAb HDD20 against BP230 and type XVII collagen, respectively. mAbs PN643, HD1-121 and PC815 were generously donated by Prof. K. Owaribe of Nagoya University, and antibody S1 193 by Prof. J. R. Stanley of the University of Pennsylvania. C20, a goat polyclonal antibody against the C-terminus of plectin, was purchased from Santa Cruz. Anti-beta-actin mAb (AC15, Sigma, St. Louis, MO) was used to confirm equal protein loading.

### Cell Culture and Immunoblot Analysis

Cell culture and immunoblot analysis was performed as previously described (Natsuga, et al., 2010). Cultured fibroblasts were obtained from skin biopsies of a normal human volunteer and the proband. Cultured fibroblasts were maintained in Dulbecco's modified Eagle's medium supplemented with 10% (v/v) fetal bovine serum. For sample preparation, cultured cells were lysed in Nonidet-40 (NP-40) containing buffer (1% NP-40, 25mM Tris-HCl (pH 7.6), 4mM EDTA, 100mM NaCl, 1mM phenylmethylsulfonyl fluoride (PMSF), and proteinase inhibitor cocktail (Sigma, St. Louis, MO)); cell debris was removed by centrifugation; and the supernatant was collected. Supernatants were boiled in Laemmli's sample buffer (Laemmli, 1970), applied to a 4–12% gradient Bis-Tris gel (Invitrogen, Carlsbad, CA), and transferred to a PVDF membrane. The membrane was incubated with PN643, HD1-121, C20 and AC15 followed by incubation with horseradish peroxidase (HRP) conjugated anti-mouse IgG (for PN643, HD1-121 and AC15) and HRP-conjugated anti-gout IgG (for C20). The blots were detected using ECL Plus Detection Kit (GE Healthcare, Fairfield, CT).

### Semi-quantitative RT-PCR Analysis

Semi-quantitative reverse transcription PCR (RT-PCR) analysis was performed as previously described (Natsuga, et al., 2010). Total RNA was isolated from cultured fibroblasts (from normal human volunteers and the proband, using RNeasy kit (Qiagen, Valencia, CA)), and first-strand cDNA was made using Superscript III reverse transcriptase (Invitrogen, Carlsbad, CA). First-strand cDNA was then amplified by PCR with primers specific for the exon boundaries flanking the rod domain of plectin as described previously (Koster, et al., 2004; Natsuga, et al., 2010). The following primers were used (Fig. 1B): 30F, 5′-CATCAGCGAGACTCTGCGGC-3′; 31R, 5′- TGCGCCTGTCGCTTTTGTGC-3′; 31F, 5′-AGCTGGAGATGAGCGCTGA-3′; 32R, 5′- TGCTGCAGCTCCTCCTGC-3′. To ensure equal loading, a housekeeping gene (GAPDH) was simultaneously amplified. The PCR products were assessed on a 2% agarose gel. The images were obtained with LAS-4000 mini (Fujifilm, Tokyo, Japan).

The medical ethics committee of Hokkaido University Graduate School of Medicine approved all of the described studies. The study was conducted according to The Declaration of Helsinki Principles. Participants gave their written informed consent.

## RESULTS

### Case Description

**Figure 2:**
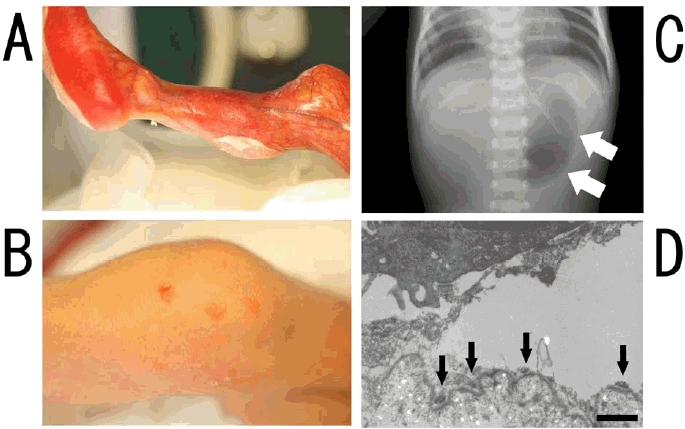
Clinical and ultrastructural features of the proband. (A) Aplasia cutis is observed on the left lower leg at birth. (B) Vesicles and erosions are scattered on the right knee. (C) Abdominal X-ray reveal single bubble sign (arrows), which indicated pyloric atresia. (D) Electron microscopy of the skin specimens from the proband reveals skin detachment within basal keratinocytes. Hemidesmosomes are hypoplastic and are observed at the base of the blisters (arrows) (Bar=1μm).

The proband was a first child of non-consanguineous Japanese parents. There was no family history of bullous diseases. He was born by cesarean section after a 39-week gestation because of non-reassuring fetal status. Clinically the proband showed extensive blistering and aplasia cutis on the extremities (Fig. 2A, B). Routine abdominal X-ray revealed a single bubble sign, suggesting the presence of PA (Fig. 2C). Generalized muscle hypotonia, dysphagia and difficulty in breathing were also observed from birth. Laboratory examination at birth revealed markedly elevated levels of creatine kinase (CK) (11,852U/L, normal value; 60-400U/L). The skeletal muscle isoform of CK (CK-MM) was 84% of total CK (CK, 2058U/L at age 12 days). Elevated levels of muscle enzymes including CK and aldolase (normal value; 1.7-5.7U/L) persisted over the course of his life (CK, 1924U/L; aldolase, 40.0U/L at age 25 days). Based on the clinical features and laboratory data, the presence of MD was confirmed. Muscle biopsy and reconstructive surgery for PA was not performed because the parents did not consent. The proband died 3 months after birth. Permission for autopsy was refused.

### Skin Separation in Basal Keratinocytes

Electron microscopy of the skin samples from the proband showed that the skin separation localized to the base of the basal keratinocytes (Fig. 2D). Hemidesmosomes were hypoplastic and found at the base of the intraepidermal split (Fig. 2D). Keratin clumps were not observed.

### *PLEC* Mutations in Exon 32

**Figure 3:**
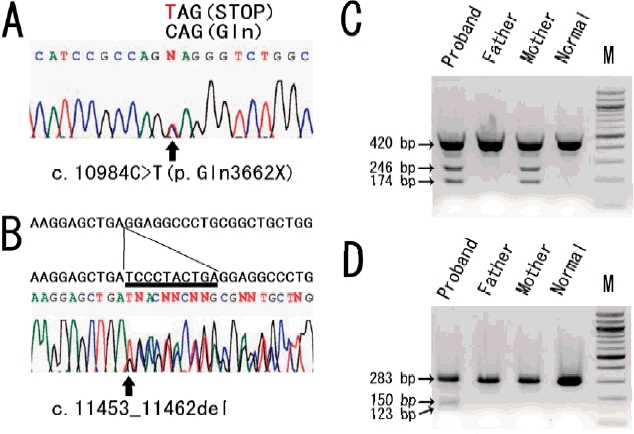
The novel *PLEC* mutations detected in the study. Maternal c.10984C>T (p.Gln3662X) (A) and de novo c.11453_11462del (B) in exon 32 were found in genomic DNA derived from the proband. A thymine substituted for a cytosine in the former mutation is indicated by the red character (A). Deleted nucleotides in the latter mutation are underlined (B). (C) c.10984C>T mutation caused the generation of a site for *BsrI* restriction enzyme. *BsrI* digestion of the 420-bp PCR product with and without the mutation resulted in a single band of 420-bp and in double bands of 246-bp and 174-bp, respectively. c.10984C>T was a maternal mutation. (D) c.11453_11462del caused the generation of a site for *BbvCI*. The 283-bp PCR product without the mutation was not digested by *BbvCI. BbvCI* digestion of the 273-bp PCR product with the deletion mutation showed two bands of 150 and 123-bp. c.11453_11462del was not detected in the parents' gDNA.

*PLEC* mutational analysis demonstrated that the proband was compound heterozygous for maternal c.10984C>T (p.Glu3662X) and de novo c.11453_11462del in exon 32, the last exon of *PLEC* (Fig. 3A, 3B, 1C). The latter mutation is predicted to result in a frameshift that causes 88-amino-acid missense sequences followed by a premature termination codon (PTC). Both of the mutations were novel. c.10984C>T was confirmed by *BsrI* restriction enzyme digestion (Fig. 3C). c.11453_11462del was also confirmed by *BbvCI* restriction enzyme digestion (Fig. 3D) and TA-cloning (data not shown). Haplotype analysis of this family using micro satellite markers excluded false paternity as well as false maternity (data not shown) to establish the de novo nature of c.11453_11463del. The father's sperm has not been tested, although it might be beneficial to exclude the small possibility of paternal germ-line mosaicism through analyzing the father's sperm for any future prenatal diagnosis. In addition, c.7587G>A (p. =) transition in exon 32 was also detected in one allele of the proband and his father. This c.7587G>A transition was found in 3 of 100 normal unrelated alleles (50 healthy Japanese individuals), and was likely a polymorphism.

### Diminished and Truncated Plectin Expression in Skin

**Figure 4:**
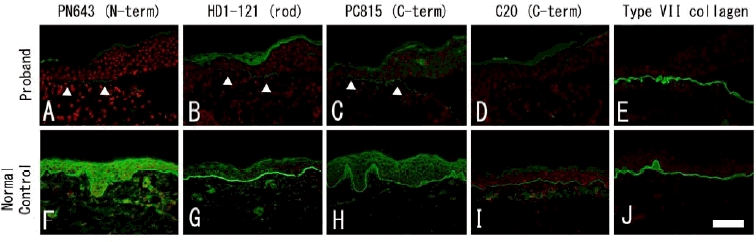
Immunofluorescence analysis of the proband's skin sample. In normal human skin, immunofluorescence shows that all of the antibodies against plectin (PN643, HD1-121, PC815 and C20) tested in this study bound to the dermal epidermal junction (DEJ) (F - I). DEJ labeling of PN643, HD1-121 and PC815 are weakly positive in the proband (A - C). In contrast, staining with C20 is negative in the proband's skin sample (D). Type VII collagen shows normal linear labeling in the proband and in the normal control (E, J). Weak labeling is indicated by arrowheads (Bar=100μm).

We performed immunofluorescence analysis of the skin specimens from the proband using several antibodies that react with molecules of the dermo-epidermal junction (DEJ). To check plectin expression patterns in the skin specimens from the proband, we used four antibodies: PN643 (N-terminal globular domain), HD1-121 (rod domain), PC815 (C-terminal globular domain) and C20 (C-terminal globular domain) (Fig. 1A). Normal human control shows bright DEJ staining of all the antibodies tested (Fig. 4F-I). DEJ labeling of PN643, HD1-121 and PC815 was markedly diminished in the skin specimens from the proband (Fig. 4A-C). Staining of C20 was absent in the proband's skin (Fig. 4D). Immunostaining for type VII collagen (Fig. 4E), laminin 332, type IV collagen, type XVII collagen, α6 and β4 integrin, and BP230 revealed normal DEJ labeling patterns (data not shown).

### Diminished and Truncated Plectin in Cultured Fibroblasts

**Figure 5:**
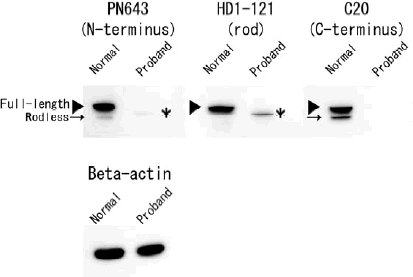
Immunoblot analysis of cultured fibroblasts from the normal human control and the proband. Immunoblot analysis of extracts from fibroblasts of the normal control and the proband by using PN643 against the N-terminal actin- binding domain, HD1-121 against the rod domain and C20 against the C-terminal plectin repeats. Rodless plectin (arrows), detected with PN643 and C20, migrates just below full-length plectin (arrowheads) in normal human fibroblasts. Using HD1-121, only full-length plectin is observed in the normal control. In contrast, fibroblasts of the proband contained smaller proteins than 500-kDa full-length plectin, the putatively truncated full-length plectin (asterisks), which was detected with PN643 and HD1-121. C20 did not react with lysates of the proband's fibroblasts. Equal protein loading was confirmed by reprobing with AC 15 (anti-beta-actin antibody).

Immunoblot analysis of lysates from normal human cultured fibroblasts revealed that two closely spaced bands, corresponding to two forms of plectin (500kDa full-length and 390kDa rodless), reacted with PN643 and C20 antibodies recognizing the N- and C-termini of plectin (Fig. 5), as previously described (Natsuga, et al., 2010). HD1-121 against the rod domain reacted only with full-length plectin in normal human fibroblasts (Fig. 5). Lysates from cultured fibroblasts from the proband showed a faint band of PN643 and HD1-121 between 500kDa and 390kD, corresponding to truncated full-length plectin. C20 failed to react with lysates from the proband's cells (Fig. 5).

### Full-length and rodless plectin transcripts are reduced in the proband's cultured fibroblasts

**Figure 6:**
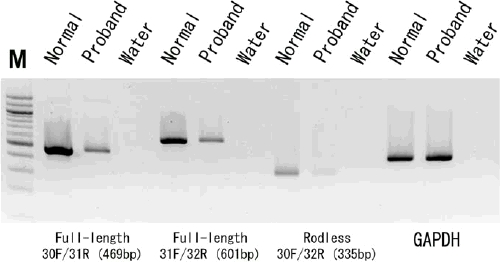
Semi-quantitative RT-PCR for full-length and rodless plectin transcripts. The quantity of full-length (30F/31R and 31F/32R) and rodless (30F/32R) plectin transcripts in the proband's cultured fibroblasts is reduced in comparison to those of the normal control. GAPDH mRNA expression was used as the loading control in these experiments. The negative control reaction (DNA-free water instead of cDNA) shows no PCR products. The molecular weight standard (lane M) is a 100-bp ladder.

Using RT-PCR, the presence of mRNA that encodes full-length or rodless plectin was demonstrated in the normal human control as well as in the proband's cultured fibroblasts (Fig. 1B, 6). Judging from the PCR analysis results, the quantity of full-length and rodless plectin transcripts was markedly reduced in the proband's fibroblasts compared with those of the normal human control (Fig. 6).

## DISCUSSION

This is the first report of EB complicated with both MD and PA. Skin detachment within basal keratinocytes was demonstrated by electron microscopy, which indicated the simplex subtype of EB. The proband's skin sample and cultured fibroblasts showed reduced and truncated plectin expression (Fig. 1D).

Both of the premature termination codon (PTC)-causing mutations detected in our case are within exon 32, the last exon of *PLEC*. Nonsense-mediated mRNA decay (NMD) is a quality-control mechanism that selectively degrades mRNAs with PTCs (Holbrook, et al., 2004). When the mRNA has a PTC more than a certain range upstream of one exon-exon junction, the transcript is down-regulated by NMD. In contrast, when PTCs are located in the last exon, NMD does not generally occur and abnormal mRNA is translated into truncated protein. However, some exceptions were described in a study in which transcripts underwent NMD despite having a PTC in the last exon (Chan, et al., 1998). Reduced amounts of full-length and rodless plectin transcripts in the proband's cells are explained by NMD, even though the PTCs of the proband are in the last exon.

One reported EBS-MD case was homozygous for a PTC-causing mutation (c.13458_13473dup) in exon 32 (Schroder, et al., 2002). c. 13458_13473dup is at the downstream of the 6^th^ plectin repeat and is predicted to cause a frameshift followed by a premature termination codon. The age of onset for MD in the patient with c.13458_13473dup was 4 years (Schroder, et al., 2002); in our case, severe muscle weakness was observed immediately after birth. This clinical difference might be explained by the length of truncated proteins identified in each patient. Compound heterozygous mutations of c.10984C>T and c.11453_11462del encode truncated plectin protein that does not include the intermediate filament (IF) binding site that was mapped to an approximately 50- amino-acid sequence between the 5^th^ and 6^th^ plectin repeat (Nikolic, et al., 1996; Rezniczek, et al., 2010). Therefore, the truncated plectin in our case might not have bound to IF including desmin in muscle tissues, which might account for the congenital muscle weakness. In contrast, the truncated plectin produced by c.13458_13473dup harbors the IF-binding site described above. Although the amount of plectin protein was slightly diminished, it may be that substantial amounts of truncated plectin with the residual IF-binding site delayed the development of muscular dystrophy and prevented pyloric atresia in the previous patient (Schroder, et al., 2002).

EBS-MD patients do not have muscular symptoms at birth, but muscle weakness appears later in their life. The type of *PLEC* mutations (PTC-causing mutations or in-frame insertions/deletions) influences the timing of MD onset (Chiaverini, et al., 2010). Also, it may be that, in most EBS-MD cases, the presence of residual rodless plectin resulting from PTC-causing mutations in exon 31 delays the onset of MD because of the remaining IF-binding site in rodless plectin.

It has been postulated that two pathologic elements are involved in the development of PA in EB patients: 1) the integrity of basement membrane and hemidesmosomes, and 2) the control of the normal process of fibrosis in the course of wound healing (Maman, et al., 1998). The sequence of events might be initiated by the separation of the intestinal mucosal layer as a result of disintegration of basement membrane and hemidesmosomes. Inflammatory responses cause massive fibrosis, which might lead to the obstruction of the intestinal lumina, especially in anatomically narrow passages, such as pylorus (Maman, et al., 1998). Previously described EBS-MD patients do not suffer from PA, which suggests that residual rodless plectin can prevent the development of PA. In our case, both full-length and rodless plectin proteins are quantitatively reduced and the shortened plectin might not have functioned normally, which might have lead to the PA phenotype.

It had been predicted that some cases of EBS-PA would develop MD, although no such case had been reported in the literature (Natsuga, et al., 2010). One possible explanation is that the poor systemic condition of EBS-PA and the limited observation period due to the patient's very short lifespan prevented the diagnosis of MD. Our data suggests that surgical correction of PA is insufficient to treat EBS-PA patients because they would most likely go on to develop MD even if they survive surgery. Therefore, we should look to develop more fundamental therapeutic options for those patients.

In summary, this study clearly shows that plectin mutations lead to both MD and PA phenotypes in an individual EBS patient.
